# Mulheres e Fibrilação Atrial: A Disparidade na Anticoagulação é apenas uma Questão de Gênero? Em Busca da Realidade Brasileira

**DOI:** 10.36660/abc.20240583

**Published:** 2024-10-22

**Authors:** Jorge Elias, Fernando Luiz Torres Gomes

**Affiliations:** 1 Vitoria Apart Hospital Serra ES Brasil Vitoria Apart Hospital – Serviço de Eletrofisiologia, Serra, ES – Brasil; 2 Hospital Universitário Cassiano Antônio Moraes Vitoria ES Brasil Hospital Universitário Cassiano Antônio Moraes, Vitoria, ES – Brasil

**Keywords:** Mulheres, Sexo, Fibrilação Atrial, Anticoagulação, Fatores de Risco, Acidente Vascular Cerebral

A discussão sobre a fibrilação atrial (FA) deve partir da premissa de que, certamente ao lado de outras condições como a obesidade, e em uma parceria particularmente prejudicial com ela, esse distúrbio do ritmo emergiu como um problema global de saúde pública, justificando uma projeção epidêmica para este século.^1^

Numerosos estudos demonstraram diferenças na apresentação da FA, incidência de comorbidades e taxas de internação entre homens e mulheres. Portanto é crucial avaliar se existem diferenças de gênero na adesão e na utilização de anticoagulantes orais, bem como nos desfechos baseados nas condições clinicas na alta hospitalar.^1–7^

Tomando como base essas considerações, a necessidade de uma publicação abordando essa questão no contexto da população brasileira se tornou evidente, embora com foco em um segmento populacional específico, como discutiremos mais adiante. Entre os diversos contextos para analisar essa disparidade entre os sexos, o setor de emergência (SE) representa um ambiente ideal para identificar possíveis lacunas no atendimento.^8^

Na edição dos ABC Cardiol, Medei et al. apresentam o primeiro estudo observacional prospectivo multicêntrico brasileiro envolvendo adultos consecutivos com FA sintomática admitidos em SE.^9^ Como principal achado, esse estudo revela uma disparidade entre os sexos quanto à indicação para terapia anticoagulante (41,8% mulheres vs. 31,3% homens) e o uso inadequado de anticoagulantes (23,5% mulheres vs. 18,2% homens).^9^ Esse resultado indica que o panorama brasileiro em relação à disparidade na anticoagulação oral entre gêneros reflete os resultados documentadas globalmente.

É imperativo analisar esses resultados à luz do paradoxo bem estabelecido de que mulheres com FA apresentam mais sintomas, pior qualidade de vida, e têm um risco maior de acidente vascular cerebral em comparação com os homens, porém são menos propensas a receber terapia anticoagulante e tratamento de controle de ritmo.^4^

A subutilização da anticoagulação oral em mulheres pode ser atribuída a múltiplos fatores. Em primeiro lugar, embora o escore CHA2DS2-VASc, incorporado nas diretrizes clínicas, reconheça o perfil de risco globalmente mais alto das mulheres, os clínicos podem aderir a dados de registro mais recentes que indicam que o sexo pode atuar como um modificador de risco em vez de um fator de risco direto, particularmente em escores de risco mais baixos.^1^ No entanto, análises de subgrupos de maior risco revelam que a subutilização de anticoagulantes em mulheres persiste.^3^

É essencial reconhecer diferenças significativas entre os sexos na epidemiologia das arritmias cardíacas e nas características eletrofisiológicas do miocárdio.^10^ Além disso, existem diferenças biológicas relacionadas ao peso corporal, à área de superfície corporal, à distribuição de água total corporal e à compartimentalização da água extracelular e intracelular.^7^ Essas características podem impactar o metabolismo dos medicamentos.^11^

Entre as diversas causas que podem explicar essas diferenças biológicas entre os gêneros, se destacam os mecanismos de coagulação que flutuam significativamente durante diferentes estados hormonais femininos em várias fases da vida (ciclo menstrual, gravidez, pós-menopausa), juntamente com fatores como a função endotelial, uso de contraceptivos e terapia de reposição hormonal.^7^ Por exemplo, o estado de hipercoagulabilidade induzido por um aumento nas citocinas inflamatórias durante o período menopausal merece atenção.^7,10^

Outras considerações críticas incluem a propensão das mulheres a receber uma "dose mais alta" das medicações em comparação com os homens — provavelmente devido a diferenças no volume de distribuição, uma "fração livre" maior dos medicamentos e variabilidade na depuração dos medicamentos — assim como sua maior sensibilidade aos efeitos dos medicamentos.^11^

Além disso, as mulheres tendem a usar um maior número de medicamentos do que os homens, aumentando assim o risco de interações medicamentosas.^7,11^ Também é importante reconhecer que "características de gênero" podem incorporar variáveis externas que atuam como fatores epigenéticos, como ambientes socioculturais, diferenças comportamentais, hábitos nutricionais, exposições ambientais, atitudes de estilo de vida e adesão à terapia.^7^

Outros pontos estruturais importantes na tomada de decisão compartilhada sobre a terapia anticoagulante incluem a adequada explicação pelo profissional de saúde dos riscos e benefícios do uso de anticoagulantes, concordância de gênero entre pacientes e médicos, e fatores não médicos que afetam os pacientes, como o custo dos medicamentos, disponibilidade nas redes de saúde pública, baixo nível de escolaridade e barreiras logísticas (como exemplo: os pacientes que habitam áreas rurais).^3^

Além disso, em algumas circunstâncias, as mulheres podem ser percebidas como mais frágeis e com menor massa corporal, levando à prescrição de doses menores dos mesmos medicamentos, o que pode comprometer a eficácia do tratamento.

Dadas as complexidades descritas, se torna compreensível como o subtratamento observado por Medei et al. contribui para um aumento do risco de acidente vascular cerebral e hospitalização entre as pacientes do sexo feminino.^9^ Além disso, em comparação com os homens, as mulheres com FA recentemente diagnosticada tendem a ser mais idosas, apresentam escores CHA2DS2-VASc mais altos, enfrentam uma carga maior^4^ de comorbidades e geralmente são tratadas em circunstâncias clínicas mais adversas. Assim, é uma questão clinicamente significativa que o alto risco enfrentado pelas mulheres não seja correspondido por respostas clínicas adequadas para mitigar esse risco aumentado.^3^

Neste ponto, é essencial mudar nossa discussão para aspectos que muitas vezes são negligenciados, mesmo no presente estudo: a influência das variáveis étnicas, raciais e socioeconômicas nas disparidades observadas no tratamento das pacientes do sexo feminino.

Duas revisões sistemáticas destacaram uma disparidade persistente na prescrição e no tipo de anticoagulante oral usado para FA, particularmente evidente ao se analisarem a raça, a etnia e o status socioeconômico dos pacientes.^2,12^

Notavelmente, no contexto da população brasileira, esses estudos sinalizam que há uma tendência preocupante de que pacientes negros têm menos probabilidade de receber prescrição de anticoagulantes orais em comparação com pacientes brancos e asiáticos, mesmo quando seus escores de risco de AVC CHA2DS2-VASc são comparáveis. Além disso, se observa uma prevalência clara do uso de varfarina nesse grupo demográfico. Vale mencionar que, mesmo em países socialmente equitativos como a Suécia, o nível de escolaridade influencia na relação entre a utilização de anticoagulantes orais diretos (DOACs) e varfarina.^2^

Existe um ponto crítico que merece ser sinalizado nesse artigo, que é a falta de consideração das questões étnicas e raciais — fundamentais em uma população diversa como a do Brasil — o que pode levar a resultados tendenciosos. O fato de o estudo ter sido conduzido principalmente em ambientes de saúde privada pode agravar tais vieses em comparação com hospitais públicos. De fato, apenas um Estado no Brasil atualmente fornece DOACs gratuitamente, o que afeta significativamente a utilização adequada da terapia anticoagulante em classes sociais mais desfavorecidas.

Além disso, é importante notar que os pacientes recebem diferentes tipos de anticoagulantes orais, dependendo se são tratados no SE, no ambiente ambulatorial ou durante internações.^2^ Esse achado reflete variações nos processos de cuidado na medicina de emergência em comparação com o cuidado ambulatorial padrão, sugerindo que pacientes desfavorecidos frequentemente recorrem ao SE devido à instabilidade clínica, indicando barreiras no acesso ao atendimento regular.

Para abordar as disparidades observadas no tratamento da FA, é crucial que futuros estudos clínicos proporcionem representação equitativa entre coortes de pacientes diversos, incorporando categorização apropriada não apenas de gênero, mas também de origens raciais, étnicas e socioeconômicas.

Os resultados de ensaios clínicos fornecem a base de evidências críticas para avaliar a segurança e a eficácia de novos medicamentos e produtos médicos. A eficácia e a segurança podem mudar entre subgrupos populacionais dependendo de diversas variáveis sub-representadas na grande maioria dos estudos realizados até o momento. Não devemos negligenciar o impacto das análises sistemáticas realizadas com base nesses estudos e os significativos vieses e impactos na prática clínica.^13^

É essencial assumir que tanto o gênero quanto outras variáveis qualitativas influenciam significativamente o acesso dos pacientes ao tratamento da FA, incluindo a prevenção de eventos tromboembólicos por meio da terapia anticoagulante.
