# Supplementary material for: Mulheres e Fibrilação Atrial: A Disparidade na Anticoagulação é apenas uma Questão de Gênero? Em Busca da Realidade Brasileira
Source: Arq Bras Cardiol. 2024 Oct 22;121(9):e20240583. [Article in Portuguese] doi: 10.36660/abc.20240583 (PMC11634200; doi:10.36660/abc.20240583)
Supplement: Translation

# Translation: Women and Atrial Fibrillation: Is the Disparity in Anticoagulation Just a Question of Gender? In Search of the Brazilian Reality

**DOI:** 10.36660/abc.20240583i

**Authors:** Jorge Elias, Fernando Luiz Torres Gomes

**Affiliations:** 1 Serra ES Brazil Vitoria Apart Hospital – Serviço de Eletrofisiologia, Serra, ES – Brazil; 2 Vitoria ES Brazil Hospital Universitário Cassiano Antônio Moraes, Vitoria, ES – Brazil

**Keywords:** Women, Gender, Atrial Fibrillation, Anticoagulation, Risk Factors, Stroke

The discussion of atrial fibrillation (AF) must begin with the premise that certainly, alongside other conditions such as obesity — and in a particularly harmful partnership with it — this rhythm disturbance has emerged as a global public health problem, warranting an epidemic projection for this century.^1^

Numerous studies have demonstrated differences in AF presentation, comorbidity burden, and admission rates between males and females. Therefore, it is critical to assess whether there are sex differences in adherence to and utilization of oral anticoagulants and outcomes based on hospital discharge status.^1–7^

Given these considerations, the necessity for a publication addressing this issue within the context of the Brazilian population has become evident, albeit focusing on a specific segment, as we will discuss later. Among the various contexts for analyzing this disparity between the sexes, the emergency department (ED) represents an optimal setting to identify potential gaps in care.^8^

In the issue of ABC Cardiol, Medei et al. present the first multicenter prospective observational study involving consecutive adults with symptomatic AF admitted to emergency departments.^9^ The central finding of this study reveals a disparity between the sexes regarding the indication for anticoagulant therapy (41.8% female vs. 31.3% male) and the inappropriate use of anticoagulants (23.5% female vs. 18.2% male).^9^ This outcome indicates that the Brazilian landscape pertaining to the disparity in oral anticoagulation between genders mirrors findings documented globally.

It is imperative to analyze these results in light of the well-established paradox that women with AF experience more symptoms, poorer quality of life, and a higher risk of stroke compared to men, yet they are less likely to receive anticoagulant therapy and rhythm control treatment.^4^

The underutilization of oral anticoagulation in women can be attributed to multiple factors. Firstly, although the CHA2DS2-VASc score, incorporated into clinical guidelines, acknowledges women's overall higher risk profile, clinicians may adhere to more recent registry data indicating that sex may act as a risk modifier rather than a direct risk factor, particularly at lower risk scores.^3^ Nonetheless, analyses of higher-risk subgroups reveal that the underutilization of anticoagulants in women persists.^3^

Significant sex differences in the epidemiology of cardiac arrhythmias and the electrophysiological characteristics of the myocardium must be acknowledged.^10^ Additionally, biological differences concerning body weight, body surface area, total body water distribution, and the compartmentalization of extracellular and intracellular water^7^ exist. These characteristics can impact drug metabolismo.^11^

Among the various causes that may account for these biological differences between genders, we emphasize the coagulation mechanisms that significantly fluctuate during different female hormonal states at various life stages (menstrual cycle, pregnancy, postmenopause), alongside factors such as endothelial function, contraceptive use, and hormone replacement therapy.^7^ For instance, the state of hypercoagulability induced by an increase in inflammatory cytokines during the menopausal period warrants attention.^7,10^

Other critical considerations include the propensity for women to be disproportionately overdosed compared to men — likely secondary to differences in the volume of distribution, a larger "free fraction" of drugs, and variability in drug clearance — as well as their heightened sensitivity to drug effects.^11^

Moreover, women tend to take a greater number of medications than men, thereby increasing the risk of drug interactions.^7,11^ It is also important to recognize that "gender characteristics" can incorporate external variables acting as epigenetic factors, such as sociocultural environments, behavioral differences, nutritional habits, environmental exposures, lifestyle attitudes, and adherence to therapy.^7^

Further points worth highlighting in shared decision-making frameworks against anticoagulation therapy include adequate clarification of the risks and benefits of anticoagulant use, gender concordance between patients and providers, and non-medical factors affecting patients, such as the cost of medications, availability in public healthcare networks, low education levels, and logistical barriers.^3^

Additionally, in some circumstances, women may be perceived as more frail and of lower body mass, leading to the prescription of lower doses of the same medications, potentially compromising treatment efficacy.

Given the complexities outlined, it becomes comprehensible how the under-treatment observed by Medei et al. contributes to an increased risk of stroke and hospitalization among female patients.^9^ Furthermore, in comparison to men, women with newly diagnosed AF tend to be older, present with higher CHA2DS2-VASc scores, experience a greater burden of comorbidity, and are generally treated under worse clinical circumstances.^4^ It is thus a clinically significant issue that the high risk faced by women is not matched by corresponding clinical responses aimed at mitigating this heightened risk.^3^

At this juncture, it is essential to pivot our discussion to aspects that are often overlooked, even in this study: the influence of ethnic, racial, and socioeconomic variables on the observed disparities in the treatment of female patients.

Two systematic reviews have highlighted a persistent disparity in the prescription and type of oral anticoagulant used for AF, particularly evident when analyzing the race, ethnicity, and socioeconomic status of patients.^2,12^

Notably, within the context of the Brazilian population, there is a concerning trend that Black patients are less likely to be prescribed oral anticoagulation compared to White and Asian patients, even when their CHA2DS2-VASc stroke risk scores are comparable. Furthermore, a clear prevalence of warfarin usage is observed in this demographic. It is worth mentioning that even in socially equitable countries like Sweden, education level influences the relationship between the utilization of direct oral anticoagulants (DOACs) and warfarin.^2^

This article underscores the critical point that failing to account for ethnic and racial considerations — which are fundamental in a diverse population such as Brazil's — can lead to biased results. The fact that the study was primarily conducted in private healthcare settings may further exacerbate such biases in comparison to public hospitals. Indeed, only one state in Brazil currently provides DOACs at no cost, significantly affecting the proper usage of anticoagulant therapy.

Additionally, it is noteworthy that patients receive different types of oral anticoagulants depending on whether they are treated in the ED, the outpatient setting, or during inpatient admissions.^2^ This finding reflects variations in care processes in emergency medicine as opposed to standard outpatient care, suggesting that underprivileged patients often resort to the ED for clinical instability, indicating barriers to accessing regular care.

To address the observed treatment disparities in AF, future clinical studies must provide equitable representation across diverse patient cohorts, incorporating appropriate categorizations of not only gender but also racial and ethnic backgrounds.

Clinical trial results provide the critical evidence base for evaluating the safety and efficacy of new medicines and medical products. Efficacy and safety may differ among population subgroups depending on several variables under-represented in the vast majority of studies conducted to date. We must not overlook the impact of systematic analyses carried out based on these studies and the significant biases and implications on clinical practice.^13^

It is essential to assume that both gender and other categorizations significantly influence patients’ access to AF treatment, including the prevention of thromboembolic events through anticoagulant therapy.
